# Chiral Symmetry Breaking in Magnetoelectrochemical Etching with Chloride Additives

**DOI:** 10.3390/molecules23010019

**Published:** 2017-12-22

**Authors:** Iwao Mogi, Ryoichi Aogaki, Kohki Takahashi

**Affiliations:** 1 Institute for Materials Research, Tohoku University, 2-1-1 Katahira, Aoba-ku, Sendai 980-8577, Japan; kohki@imr.tohoku.ac.jp; 2 Polytechnic University, Sumida-ku, Tokyo 130-0026, Japan; AOGAKI.Ryoichi@nims.go.jp

**Keywords:** surface chirality, magnetoelectrolysis, magnetohydrodynamic vortex, chiral symmetry breaking, copper film, alanine

## Abstract

Magnetoelectrolysis (electrolysis under magnetic fields) produces chiral surfaces on metal thin films, which can recognize the enantiomers of amino acids. Here, the chiral surface formation on copper films is reported in magnetoelectrochemical etching (MEE) at 5T with chloride additives. In the absence of additives, the surface chirality signs of MEE films depended on the magnetic field polarity. On the contrary, the MEE films prepared with the additives exhibited only d-activity in both magnetic field polarities. This result implies that the specific adsorption of chloride additives induces the chiral symmetry breaking for the magnetic field polarity.

## 1. Introduction

Recent findings of redox potentials on the mineral surfaces in deep-see hydrothermal fields imply that electrochemical reactions would make a considerable contribution to the molecular evolution of the early earth [1,2]. If such mineral surfaces have chirality, the enantioselective electrochemical reactions could lead to homochiral molecular evolution. Hence, the chiral surface formation in deposition and dissolution processes is significant not only in catalytic applications in pharmaceutical synthesis but also in biomolecular homochirality.

Our previous papers showed that electrodeposition under magnetic fields (magnetoelectrodeposition; MED) produces chiral surfaces on silver and copper films, which can recognize enantiomers of glucose, amino acids, and tartaric acid [3,4,5]. Moreover, it was found that magnetoelectrochemical etching (MEE) induces surface chirality on copper films [6].

**Figure 1:**
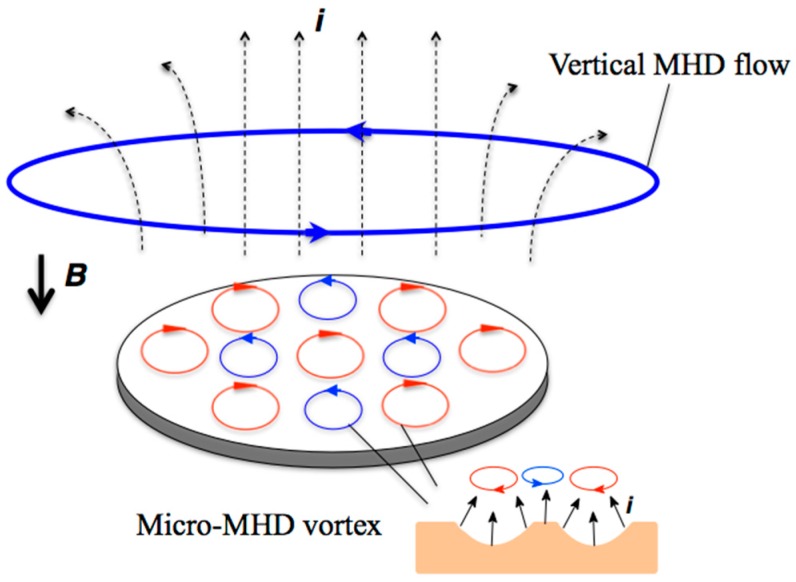
MHD effects in magnetoelectrochemical etching with vertical magnetic fields ***B***. The Lorentz force acting on the ionic current ***i*** causes two types MHD flows; macroscopic vertical MHD flow around the electrode edge and micro-MHD vortices around the non-equilibrium fluctuations (pits) on the deposit surface.

When a magnetic field ***B*** is imposed perpendicularly on a working electrode in an electrochemical cell, the Lorentz force acting on ionic currents ***i*** causes two types of magnetohydrodynamic (MHD) convections [7], as shown in Figure 1 for the MEE process. The first one is termed a vertical MHD flow. This is a macroscopic flow around the electrode edge, in which the ionic currents are not parallel to the magnetic field. The second one is micro-MHD vortices excited locally around pits caused by the non-equilibrium fluctuation in etching processes. The micro-MHD vortices could affect the growth of chiral screw dislocations and thus contribute to the chiral surface formation. The micro-MHD vortices consist of both clockwise and anticlockwise flows, such that the adjoining flows never conflict with each other. The vertical MHD flow breaks such symmetrical micro-MHD state: The cyclonic micro-MHD vortices are stable, whereas anticyclonic ones become unstable [8,9]. The stable micro-MHD vortices could contribute to the formation of screw dislocations. The direction of vertical MHD flow depends on the magnetic field polarity. Thereby, the surface chirality sign should be opposite when the magnetic field is reversed, representing the odd magnetic field dependence of chirality [10].

Specific adsorption of chloride ions on the copper MED films had a considerable influence on surface chirality. At a certain concentration of chloride additives, the MED films exhibited only l-active chirality in both magnetic field polarities [11]. This means that the chiral symmetry is broken for the magnetic field polarity. This fact is of great interest in connection with the homochirality of biomolecules. To explore the experimental conditions for the chiral symmetry breaking, in this study we have examined the chloride additive effects on the surface chirality in the MEE of copper films instead of the MED. 

## 2. Results and Discussion

### 2.1. Surface Morphology of the MEE Films

**Figure 2:**
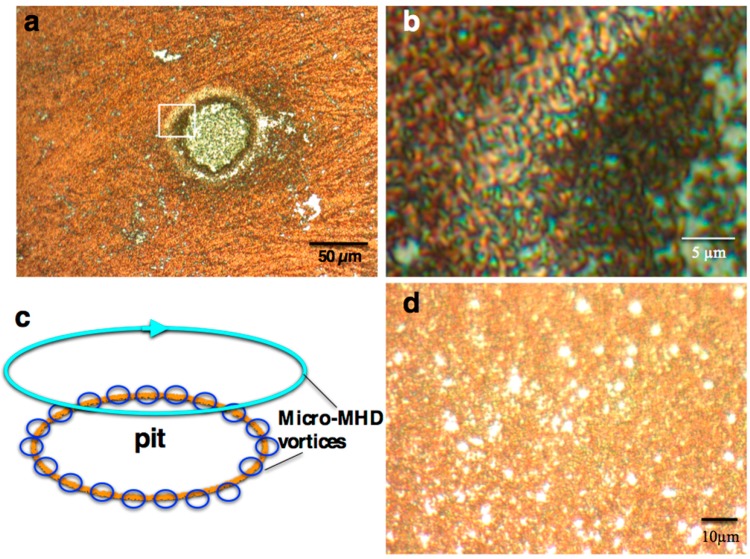
Microphotographs of MEE film surfaces prepared at a deposition current of 23 mA·cm^−2^. (**a**) +5T-film around a pit; (**b**) zoom-in photograph at the edge of pit (the rectangle part in (**a**)); (**c**) schematic of multi-scale micro-MHD vortices around a pit; (**d**) 0T-film.

The surface morphology of MEE films reflects the aspects of micro-MHD vortices. Figure 2a shows a microphotograph of MEE +5T-film, in which there is a large circular pit with a 60 µm diameter. Figure 2b shows a zoom-in photo at the edge of pit (the rectangle part in Figure 2a), in which network structures are propagating well. The typical surface morphology caused by the micro-MHD vortices is micro-circles [12] or network structures [13]. The morphologies in Figure 2a,b indicate the existence of multi-scale micro-MHD vortices, as shown in Figure 2c. Such multi-scale vortices were suggested by the visualization study of micro-MHD vortices using light-reflecting guanine micro-crystals as a fluid tracer [14]. The surface morphologies of the MEE films prepared with chloride additives were similar to those without additives. On the other hand, the surface morphology of 0T-film has a lot of pits (Figure 2d), which have various sizes and are randomly distributed because of the lack of vortex organization. 

### 2.2. Surface Chirality of the MEE Films

**Figure 3:**
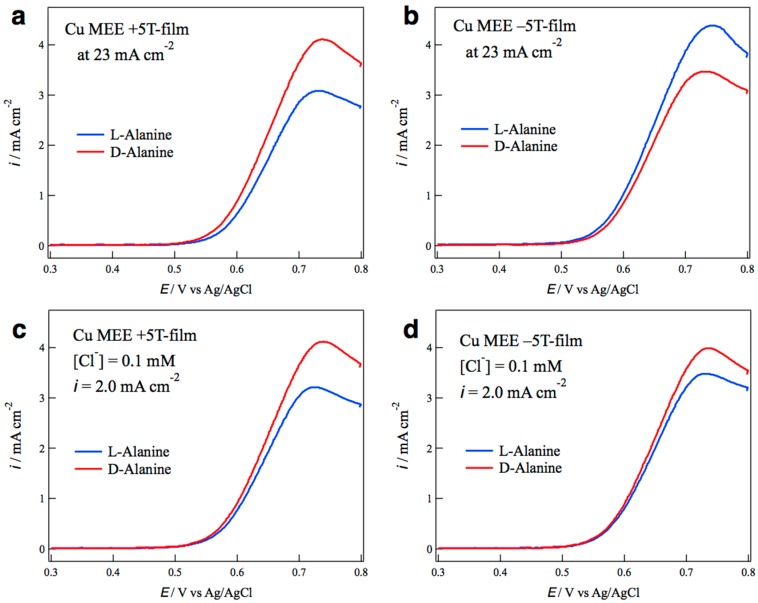
Chiral behaviors of the MEE film electrodes for the oxidation of alanine enantiomers. (**a**) +5T-film prepared at a deposition current of 23 mA·cm^−2^ without KCl; (**b**) −5T-film at 23 mA·cm^−2^ without KCl; (**c**) +5T-film at 20 mA·cm^−2^ with 0.1 mM KCl; and (**d**) −5T-film at 20 mA·cm^−2^ with 0.1 mM KCl.

The surface chirality of MEE film was estimated by the voltammetric measurements of alanine enantiomers on the MEE film electrodes, according to which the MEE films prepared under parallel and antiparallel 5T magnetic field to the ionic currents are called +5T-film and −5T-film, respectively. Figure 3a shows voltammograms of l- and d-alanines on the MEE +5T-film electrode prepared at a deposition current of 23 mA·cm^−2^ without chloride additive. The current peaks around 0.7 V correspond to the oxidation of alanine molecules, and the peak current of d-alanine is greater than that of l-alanine. This represents that the +5T-film has d-active chirality. On the other hand, Figure 3b shows that the −5T-film has l-active chirality. These facts indicate that the surface chirality of MEE films depends on the magnetic field polarity, and such odd magnetic field dependence of chirality can be expected from the effect of vertical MHD flow, as shown in Figure 1. The chloride additives appreciably changed the surface chirality of the MEE ±5T-films. Figure 3c,d shows the chiral electrode behaviors of the MEE +5T- and −5T-films, respectively, in which both films were prepared at the deposition current of 20 mA·cm^−2^ with 0.1 mM KCl. It is surprising that both +5T-film and −5T-film electrodes exhibit d-active chirality. This result suggests that the chloride additives break the odd magnetic field dependence of chirality.

The surface chirality was evaluated by the enantiomeric excess (*ee*) ratio defined as

**Figure 4:**
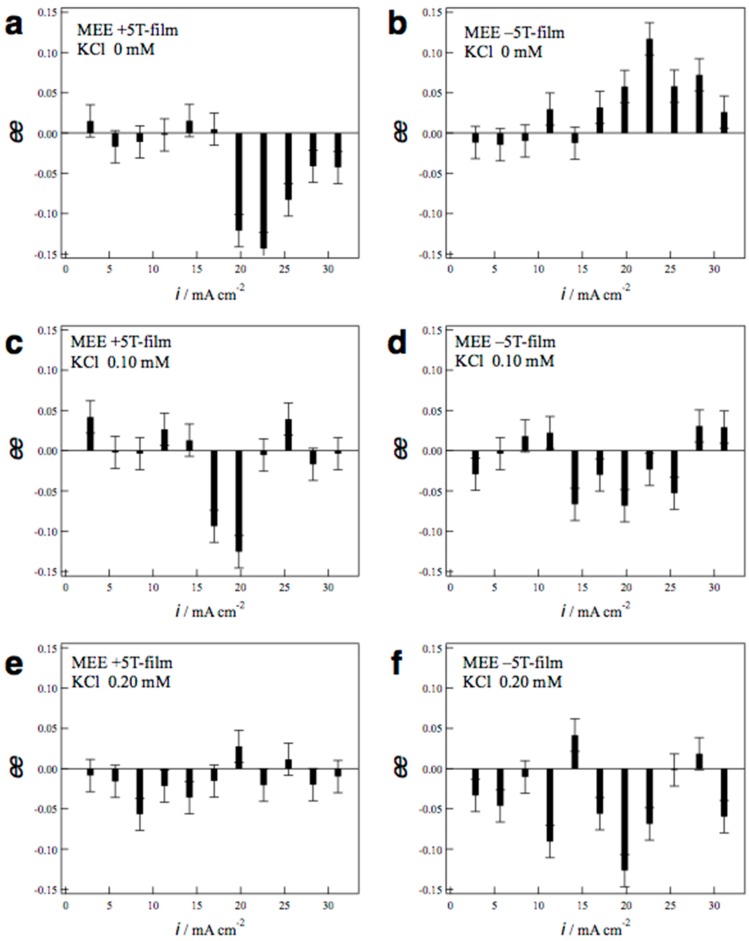
The *ee* ratio profiles of the MEE films versus the deposition currents. (**a**) +5T-film prepared without KCL; (**b**) −5T-film without KCl; (**c**) +5T-film with 0.10 mM KCl; (**d**) −5T-film with 0.10 mM KCl; (**e**) +5T-film with 0.20 mM KCl; and (**f**) −5T-film with 0.20 mM KCl. The error bars reflect the values of multiple experiments for each condition.

*ee* = (*i*_p_^L^ − *i*_p_^D^)/(*i*_p_^L^ + *i*_p_^D^), (1)

where *i*_p_^L^ and *i*_p_^D^ represent the peak currents of l- and d-alanines, respectively. The positive sign of *ee* ratio represents l-activity, and the negative sign represents d-activity. Figure 4 shows the *ee* ratio profiles (the *ee* ratio versus the deposition current) for the MEE ±5T-films prepared with KCl additives of 0, 0.1, and 0.2 mM. In the absence of KCl, the +5T-film shows d-activity in the current range of 20–30 mA·cm^−2^ (Figure 4a), whereas the −5T-film shows l-activity in the same current range (Figure 4b), representing clear odd magnetic field dependence. On the other hand, in the case of 0.1 mM KCl additives, both the +5T- and −5T-films exhibit d-activity in the current range of 15–20 mA·cm^−2^ (Figure 4c,d). Moreover, in the case of higher KCl concentration of 0.2 mM, the +5T-film shows slight d-activity in the range of 5–15 mA·cm^−2^ (Figure 4e), and the −5T-film shows clear d-activity in the almost whole current range (Figure 4f). At both KCl concentrations, the *ee* profiles represent the chiral symmetry breaking for the magnetic field polarity. 

Similar chiral symmetry breaking was observed in the MED of copper with chloride additives [11], where both +5T-film and −5T-films exhibit l-activity in the KCl concentrations of 0.13–0.20 mM. It should be noted that the MED with chloride shows l-activity, and the MEE with chloride shows d-activity. The current polarity is opposite between deposition and etching, hence the surface chirality exhibits odd current dependence between MED and MEE. This is in contrast to the chiral symmetry breaking for the magnetic field polarity.

The chloride additive effects on the electroplating of copper have well been studied [15,16,17]. Some remarkable effects, e.g., smooth surfaces and the acceleration of deposition reactions, arise from the specific adsorption of chloride ions on the deposit surfaces. Yanson and Rost [15] reported that the chloride adsorption leads to the straightening of growing steps and the changes in the step orientations. Their results suggest that the chloride adsorption could affect the screw dislocation growth: The straightening of growing steps would disturb the formation of screw dislocations, and the changes in the step orientation might cause the chirality change. Such adsorption effects on the growing steps are the one crucial point for the understanding of chiral surface behavior. Furthermore, the specific adsorption could disturb the self-organized state of micro-MHD vortices. However, it is difficult to understand the same chiral sign for both magnetic field polarities within the framework of simple MHD structures like Figure 1. The self-organized state of multi-scale micro-MHD vortices like Figure 2c might be another crucial point. In future studies, further MEE experiments with other kinds of additives under various magnetic fields are necessary to elucidate the chiral symmetry breaking.

## 3. Materials and Methods

### 3.1. Electrochemical Cell and Etching Procedures

The electrochemical cell consists of a conventional three-electrode system; a polycrystalline Pt disc working electrode with a diameter of 3 mm, a Cu plate counter electrode, and a Ag|AgCl|3 M (M = mol dm^−3^) NaCl reference electrode. Before the etching processes, Cu films were prepared with a thickness of approximately 300 nm on the working electrode by electrodeposition in a 50 mM CuSO_4_ + 0.5 M H_2_SO_4_ aqueous solution in the absence of magnetic field. The electrochemical etching of the Cu films was conducted in the CuSO_4_ + H_2_SO_4_ solution containing 0.10–0.25 mM KCl additives in galvanostatic conditions with various constant currents of 3.0–32 mA·cm^−2^. The passing charges were 0.80 C cm^−2^ in the electrodeposition and 0.40 C cm^−2^ in the MEE process, and the MEE film thickness was approximately 150 nm. 

### 3.2. MEE Procedures

The electrochemical cell was placed in the bore of cryocooled superconducting magnet (Sumitomo Heavy Industries Ltd., Shinagawa-ku, Japan), and a magnetic field of 5T was imposed perpendicularly to the working electrode surface and parallel (+5T) or antiparallel (−5T) to the ionic currents. The experimental configuration of MEE was schematically described in the previous paper [6]. The surface morphologies of the MEE films were observed by a digital photomicroscope (model VHX-100, Keyence, Osaka, Japan) just after the preparation.

### 3.3. Estimation of Surface Chirality

To examine the chirality, the fresh MEE films just after the preparation were employed as electrodes, and voltammograms of alanine enantiomers were measured after the pretreatment, which was a potential sweep between −0.3–0.3 V in a 0.1 M NaOH aqueous solution [4]. The voltammetric measurements of alanine were conducted in the absence of magnetic field in a 20 mM alanine + 0.1 M NaOH aqueous solution with a potential sweep rate of 10 mV/s. The *ee* values were estimated from more than three times experiments at each deposition current.

## 4. Conclusions

We have shown that the chloride additives appreciably affect the chiral surface formation of the copper MEE films and break the odd magnetic field dependence of chirality. In contrast, the comparison between the MEE and MED with chloride additives indicates the odd current dependence of chirality. These facts are of great interest in connection with the formation of chiral reaction fields for the homochiral molecular evolution.
